# Immature teratoma mimicking pulmonary stenosis: a case report

**DOI:** 10.1186/s13256-018-1651-x

**Published:** 2018-05-09

**Authors:** Dardan Koçinaj, Xhevdet Krasniqi, Aurora Bakalli

**Affiliations:** 1 Medical Faculty, University of Gjakova, Gjakova, Republic of Kosovo; 2 Medical Faculty, University of Prishtina, Prishtina, Republic of Kosovo; 3 UCCK, Rrethi i Spitalit n.n., 10000 Prishtina, Republic of Kosovo

**Keywords:** Mediastinal mass, Immature teratoma, Echocardiography, Magnetic resonance

## Abstract

**Background:**

Immature teratoma in a mediastinal location is a rare disease that might present as a valve pathology. Germ cell tumors with mediastinal locations account for up to 6% of immature teratoma cases. We present a case of an immature teratoma located primarily in the anterior mediastinum that manifested solely through symptoms of pulmonary stenosis.

**Case presentation:**

We report a case of a 20-year-old white man with an immature teratoma who presented with progressive exertional dyspnea. During a cardiac examination, an ejection systolic murmur was observed, and echocardiography findings at an Emergency Centre revealed high velocity flow at the level of the pulmonary artery, indicating pulmonary stenosis. He was hospitalized in our Cardiology Department for further investigation. A chest X-ray revealed a mediastinal mass, and repeated echocardiography indicated the presence of a large mediastinal mass compressing his main pulmonary artery. Magnetic resonance imaging confirmed the tumor in the mediastinum, and a histopathological diagnosis of immature teratoma was established following biopsy.

**Conclusion:**

Immature teratoma causing cardiac-related complaints might shift the diagnosis toward cardiovascular diseases, thus requiring prompt examination by standard and sophisticated methods to clarify the diagnosis.

## Background

Germ cell tumors account for up to 6% of all mediastinal tumors. They are more common in men and young adults. Common tumors, such as teratomas, are usually located in the anterior part of the mediastinum. They grow slowly, but when they become large they can compress nearby organs and cause symptoms such as dyspnea, chest pain, or coughing [1, 2]. We present the case of a young man with a primary mediastinal immature teratoma that caused compression of the main pulmonary artery, mimicking pulmonary stenosis.

## Case presentation

A 20-year-old white man, a student, presented to our clinic with a history of exertional breathlessness, non-productive cough, and fatigue over the previous 2 months. He received ambulatory treatment with antibiotics for his cough that was unsuccessful in relieving his symptoms.

**Fig. 1:**
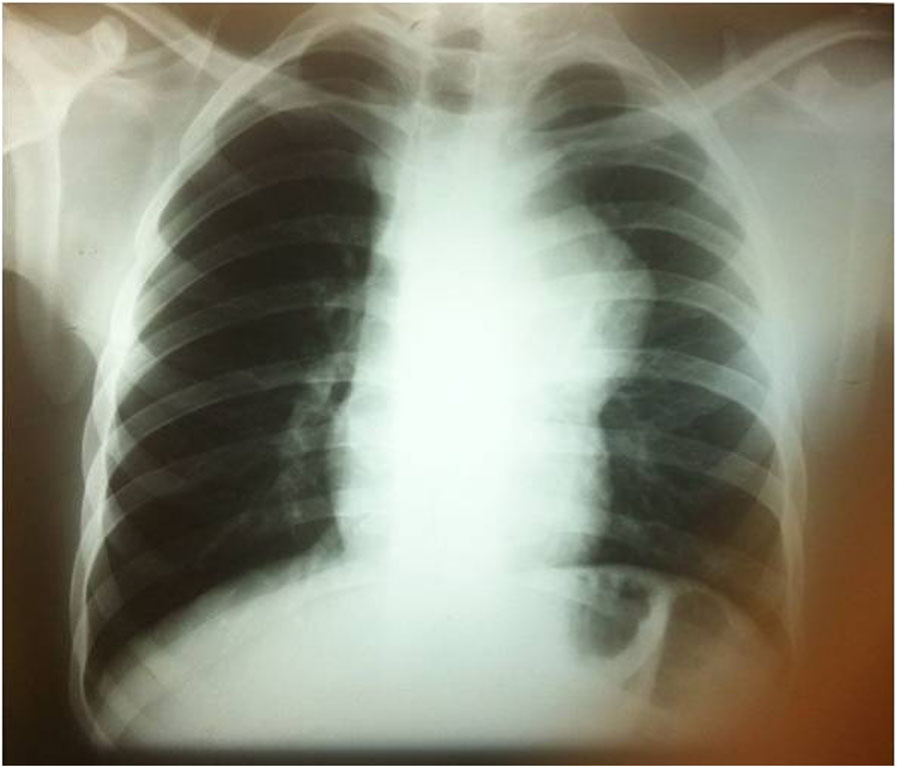
Posterior-anterior chest X-ray demonstrating mediastinal widening

A physical examination on admission revealed an ejection systolic murmur at the left sternal border with no radiation. His heart rate was 130 beats per minute (bpm), and his blood pressure was 110/60 mmHg. His lung fields, lymph nodes, and testicles were unremarkable during an examination. A neurological examination, routine blood analysis, and urine analysis were approximately within normal ranges. A resting 12-lead electrocardiogram demonstrated sinus tachycardia. A posterior-anterior native chest radiograph indicated mediastinal widening at the level of the pulmonary cone, which is indicative of a mediastinal mass (Fig. 1).

**Fig. 2:**
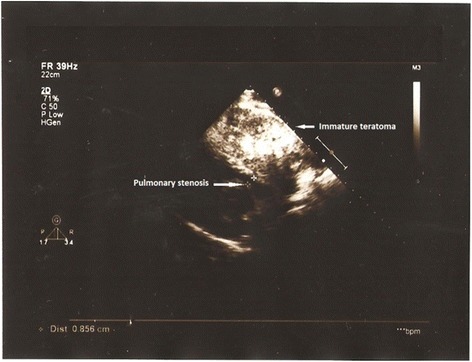
Two-dimensional parasternal short-axis view demonstrating the compression of the pulmonary artery by the mediastinal mass

Transthoracic echocardiography (TTE) in the parasternal view revealed a large extracardiac mass that was compressing the pulmonary artery (Fig. 2).

**Fig. 3:**
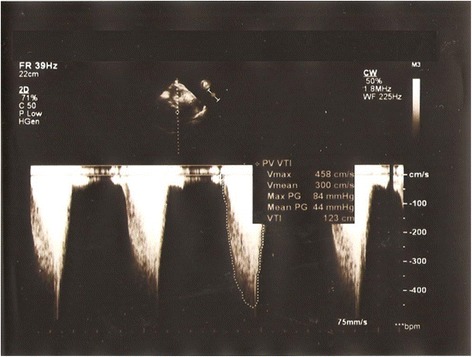
Continuous wave Doppler of the main pulmonary artery indicating a mean pressure gradient of 44 mmHg at the site of compression

A continuous wave Doppler examination showed an increased velocity (Vmax 458 cm/second, Vmean 300 cm/second) with a peak systolic gradient of 84 mmHg and a mean gradient of 44 mmHg in the pulmonary artery at the site of compression (Fig. 3).

**Fig. 4:**
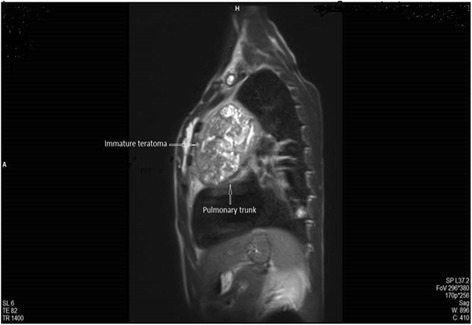
Magnetic resonance imaging showing the compression of the main pulmonary artery

**Fig. 5:**
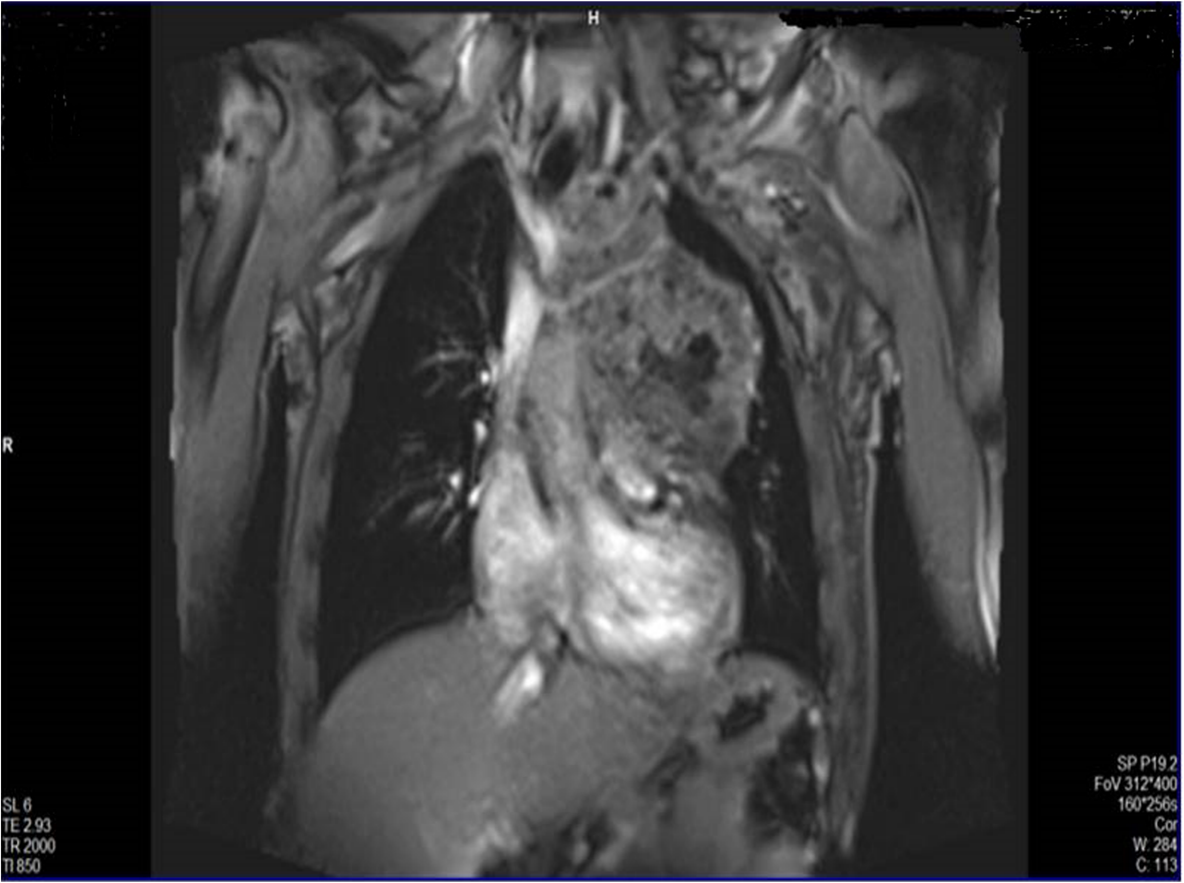
Magnetic resonance imaging of the chest showing the anterior mediastinal mass near the pulmonary artery

To better define the characterization, location, and operability of the tumor, magnetic resonance imaging (MRI) of his chest was performed, which confirmed the presence of an anterior mediastinal mass compressing his main pulmonary artery (Figs. 4 and 5).

The results of a gonadal examination and scrotal ultrasonography were normal. A biopsy confirmed an immature germ cell teratoma. Histological sections stained with hematoxylin-eosin showed immature mesenchyme as a sparsely cellular, loose, primitive mesenchymal tissue with mitotic figure. In addition, a neuroepithelial component was present in the form of rosettes. Two treatment methods were used due to the long survival periods that can be expected with them: following surgical resection, an appropriate course of chemotherapy in our Oncology department led to a decrease in tumor bulk and an improvement in our patient’s symptoms (dyspnea) during 6-month follow-up. He experienced no symptoms while receiving chemotherapy.

## Discussion

Immature teratoma located primarily in the anterior mediastinum might present as a valve pathology manifesting solely through symptoms of pulmonary stenosis.

Primary germ cell tumors account for approximately 15% of anterior mediastinal tumors in adults and children [3]. Germinal cell tumors (GCTs) are usually diagnosed in men and are generally located in the gonad. They are divided into two groups: seminomas and non-seminomas (embryonal carcinoma, mature and immature teratomas, yolk sac tumors, choriocarcinoma, and mixed germ cell tumors) [4]. In up to 6% of cases, these tumors occur in the mediastinum. Extragonadal locations, such as the peritoneal space, lung, liver, and hypophysis, are rare but are associated with poor prognoses.

Immature (germ cell) teratoma is mostly found in men, specifically in the mediastinum (anterior); there, it affects the pulmonary artery flow and causes stenosis. Mediastinal localization is very rare. It is only observed in 1% of all mediastinal teratomas, but mediastinal tumors are generally symptomatic. Thus, patients may present with dyspnea, cough, and chest pain [5]. The physical examination of our patient revealed a loud systolic murmur along the left sternal border, which was more intense during inspiration. A loud systolic murmur diagnosed during a physical examination in the chest near the left sternal border may be caused by valvular anomalies. The narrowing of the pulmonary artery may be the cause of a systolic murmur or elevated pressure in the right ventricle; however, the diagnosis must be clearly determined through further examinations [6–8].

Echocardiography (Doppler) enables the investigation of cardiac and/or paracardiac structures and may enhance the visibility, as well as the severity, of great vessel compressions [9]. In our case, we were able to visualize the extracardiac mass, quantify the echocardiography Doppler investigation, and determine high velocities at the level of the pulmonary artery. Further investigations, such as MRI, showed that the tumor contained multiple cysts and markedly compressed the main pulmonary artery. A biopsy served as the gold standard for the final diagnosis.

Teratomas are commonly classified using the Gonzalez–Crussi grading system: 0, mature; 1, immature, probably benign; 2, immature, possibly malignant (cancerous); and 3, true malignant. In the case of the latter, additional cancer staging applies.

The treatment of choice is complete surgical removal. In cases of malignant teratomas, this treatment is followed by chemotherapy. Teratomas that are located in surgically inaccessible locations (that is, complex) are likely to be malignant (late discovery and/or treatment) and are sometimes treated with chemotherapy first [9, 10].

Regarding prognoses, when complete resection is combined with chemotherapy, a long survival period can be expected.

## Conclusions

Echocardiography is the method of choice for the initial approach in determining the nature of heart murmurs. MRI is a valuable imaging method that complements the diagnosis of infiltrative masses that might cause the compression of cardiac structures and mimic valvular heart disease, thus facilitating further management and a treatment plan.

## Abbreviations

bpm: Beats per minute
GCT: Germinal cell tumors
MRI: Magnetic resonance imaging
TTE: Transthoracic echocardiography
